# Advancing the reporting of pediatric EEG data: Tools for estimating reliability, effect size, and data quality metrics

**DOI:** 10.1016/j.dcn.2024.101458

**Published:** 2024-09-28

**Authors:** Wenyi Xu, Alexa D. Monachino, Sarah A. McCormick, Emma T. Margolis, Ana Sobrino, Cara Bosco, Cassandra J. Franke, Lauren Davel, Michal R. Zieff, Kirsten A. Donald, Laurel J. Gabard-Durnam, Santiago Morales

**Affiliations:** a Department of Psychology, University of Southern California, USA; b Department of Psychology, Northeastern University, USA; c Department of Pediatrics and Child Health, University of Cape Town, USA

**Keywords:** Electroencephalogram (EEG), Data quality metrics, Event-related potentials (ERPs), Reliability, Standard Measurement Error (SME), Effect sizes

## Abstract

EEG studies play a crucial role in enhancing our understanding of brain development across the lifespan. The increasing clinical and policy implications of EEG research underscore the importance of utilizing reliable EEG measures and increasing the reproducibility of EEG studies. However, important data characteristics like reliability, effect sizes, and data quality metrics are often underreported in pediatric EEG studies. This gap in reporting could stem from the lack of accessible computational tools for quantifying these metrics for EEG data. To help address the lack of reporting, we developed a toolbox that facilitates the estimation of internal consistency reliability, effect size, and standardized measurement error with user-friendly software that facilitates both computing and interpreting these measures. In addition, our tool provides subsampled reliability and effect size in increasing numbers of trials. These estimates offer insights into the number of trials needed for detecting significant effects and reliable measures, informing the minimum number of trial thresholds for the inclusion of participants in individual difference analyses and the optimal trial number for future study designs. Importantly, our toolbox is integrated into commonly used preprocessing pipelines to increase the estimation and reporting of data quality metrics in developmental neuroscience.

## 1 Background

Electroencephalography (EEG) measures brain activity across the lifespan and is widely used for studying brain function in various domains such as neuroscience, psychology, and psychiatry. Its non-invasive nature, high cost-effectiveness, mobility, scalability, and temporal resolution have made EEG a powerful tool in developmental neuroscience research (Buzzell et al., 2023, Lopez et al., 2023, Troller-Renfree et al., 2021). Numerous EEG studies serve to characterize the development of various cognitive and affective phenomena (Morales et al., 2022), provide unique measures of individual differences (Sanchez-Alonso and Aslin, 2020), and evaluate the effectiveness of interventions (e.g., Buzzell et al., 2023; Debnath et al., 2020; Troller-Renfree et al., 2022). Beyond clinical settings, EEG research also provides neurobiological correlates that have policy implications in areas such as education, health and family policy (e.g., Apochi, 2023; Pavlakis et al., 2015; Troller-Renfree et al., 2022). These clinical and policy implications underscore the importance of utilizing reliable EEG measures and enhancing the reproducibility of EEG studies. In addition, although pediatric EEG has been mostly used in relatively small samples, researchers are becoming increasingly aware of EEG’s potential to advance neurodevelopmental research further when integrated with large datasets (Norton et al., 2021). Studies such as the Autism Biomarkers Consortium for Clinical Trials (ABC-CT) (McPartland et al., 2020), the Youth Of Utrecht (YOUth) Study (Onland-Moret et al., 2020), and the HEALthy Brain and Cognitive Development (HBCD) Study (Fox et al., 2024, Volkow et al., 2021) employ EEG techniques across multiple sites to identify biomarkers to study neurodevelopmental disorders as well as measure cognitive and emotional development in large-scale research. These studies utilize EEG to capture changes, maturation, and learning over time, thus contributing to the understanding of developmental processes.

The increasing amount of large, longitudinal EEG datasets underscores the necessity for automated EEG pipelines. Such necessity has led to the development of several publicly available EEG preprocessing pipelines, such as PREP (Bigdely-Shamlo et al., 2015), MADE (Debnath et al., 2020), and HAPPE (Gabard-Durnam et al., 2018, Monachino et al., 2022). These standardized pipelines provide distinct advantages in automating adult and pediatric data processing. Compared to manual visual inspection, these automated pipelines offer a more time-efficient alternative and provide a standardized method for removing artifacts, especially in large sample sizes. Additionally, they eliminate inter-rater differences that commonly arise with preprocessing steps that require visual inspection and subjective judgments (Leach et al., 2020). Because of these advantages, automated preprocessing approaches are becoming a common way to preprocess and analyze EEG data, even among studies with relatively small samples.

The increased use of EEG for studying neurodevelopment with policy and clinical implications, along with its expansion into larger sample sizes and automated pipelines, also highlights the importance of evaluating and reporting critical data characteristics, such as the psychometric properties of EEG measures. In addition, as research employing EEG shifts focus from characterizing broad group differences to delving into the nuances of individual differences in brain function, the relevance of considering the psychometric properties of EEG metrics becomes increasingly important (Lopez et al., 2023). Understanding individual differences in brain function through EEG requires a robust framework that accounts for the reliability and validity of EEG metrics. These psychometric properties are crucial because they ensure that the EEG measures accurately capture the individual variability in brain activity.

## 2 Current study

Despite the importance of data quality metrics for understanding data characteristics as well as individual differences, reports on the reliability and validity of EEG measures in pediatric populations are scarce. The lack of reporting and use for these metrics in EEG research may partly be because they require additional preprocessing, which can be computationally demanding and is not provided by most preprocessing packages. Because of this, adding data quality metrics as a standard output of the automated preprocessing pipelines will likely increase the use of these metrics in research moving forward. Here, we remove this barrier to access by introducing the Reliability, Effect size, And Data quality In EEG (READIE) Toolbox, written in MATLAB, and integrating it with automated software to provide automated and straightforward computation of multiple key psychometric and data quality parameters. The READIE Toolbox facilitates the estimation per dataset of reliability, effect size, and data quality measures (e.g., standardized measurement error; SME; Luck et al., 2021) with a user-friendly interface that facilitates both computing and interpreting these measures. In addition, the READIE Toolbox provides subsampled internal consistency reliability and effect size in increasing numbers of trials. Collectively, these estimates can guide the determination of the required number of trials for identifying reliable measurements and significant effects. They can help establish the minimum threshold of trials necessary for participant inclusion and provide guidance on the optimal number of trials for the design of future studies.

Our manuscript has the following aims: First, we introduce the READIE Toolbox, designed to enhance the reporting of data quality metrics for widely-used automated EEG pipelines for large-scale developmental studies, by reviewing the data quality metrics currently provided in the READIE Toolbox. These metrics include internal consistency reliability, effect sizes, and Standard Measurement Error (SME). Next, we demonstrate the capabilities of the READIE Toolbox using three EEG tasks (visual-evoked potential [VEP] task, Resting State task, and passive-viewing face processing task) collected from the pediatric population. Finally, we discuss the limitations and future directions for the toolbox.

## 3 Metrics chosen

The READIE Toolbox includes three data quality metrics: internal consistency reliability, within-person effect size, and SME. Here, we explain the details of these three metrics.

### 3.1 Internal consistency reliability

Reliability broadly evaluates the consistency and stability of the measurements used and, for a specific construct, reflects the inherent data quality (Cronbach, 1957). Specifically, for EEG studies, internal consistency reliability assesses the degree to which trials or epochs are correlated with one another. Given that EEG studies rely on stable individual measures of brain function to relate to individual measures of cognitive, affective, and sensory processing and performance (Parsons et al., 2019), evaluating reliability measures of those brain estimates would be a prerequisite for identifying potential brain-behavior relations. Similarly, in the context of large-scale longitudinal studies (e.g., HBCD; Volkow et al., 2021), EEG measures with adequate reliability would be necessary to evaluate associations between individual differences in brain functions and external factors such as environments, experiences, and exposures. In addition, as noted by Vul et al., (2009) and Parsons et al., (2019), the reliability of measures limits the strength of the association that can be determined between individual difference measures (Nunnally Jr., 1970). Thus, considering and optimizing internal consistency reliability estimates are crucial to draw robust conclusions on individual differences such as biomarkers or other brain-behavior relations.

Importantly, given that internal consistency reliability is a property of the test scores rather than the test itself, there's no simple conclusion on whether "a test is reliable or unreliable" (Wilkinson, 1999, p. 596). In the context of EEG, this suggests that the internal consistency of EEG data is not a universal trait but varies within each sample and paradigm. In other words, internal consistency reliability reflects a characteristic of the data at hand and the estimated measures (e.g., for that population) rather than an inherent characteristic of the EEG measure (e.g., ERP component; Clayson and Miller, 2017). Therefore, it is crucial to report internal consistency reliability in every study, as they can vary by several factors such as sample characteristics (e.g., age, Morales et al., 2022; clinical diagnosis, Baldwin et al., 2015), EEG recording procedures, or task environments (Clayson and Miller, 2017). For example, in a study of 4- to 9-year-olds, Morales and colleagues (2022) found that internal consistency reliability was higher for older than younger children for error monitoring measures. Similarly, Baldwin and colleagues (2015) reported a different internal consistency reliability for the control group compared to the group with clinical diagnoses. These results suggest differences in internal consistency reliability across various participant groups (e.g., age; clinical vs. typical) in each study, highlighting the necessity for studies to report the reliability of their EEG measures on a study and population basis.

In addition to obtaining an overall measure of the internal consistency reliability of the measure for the sample, estimates can be computed in an increasing number of trials. This can provide guidance on thresholds for the minimum number of trials required for reaching acceptable levels of internal consistency to include participants in analyses of individual differences. Previous research has proposed thresholds for reliability (e.g., acceptable [.60], good [.70–.80], and excellent [.90]; Clayson and Miller, 2017; Meyer et al., 2014), but these vary across studies. In addition, providing changes in internal consistency reliability as a function of increasing numbers of trials can inform optimization efforts within a dataset’s analyses and inform decisions about the number of trials for future study designs. However, it is worth noting that not all paradigms should aim for high internal consistency reliability across all trials. For example, for tasks that involve conditions or changes in the measurement construct over time (e.g., learning or habituation tasks), the internal consistency threshold should be set differently for different conditions or phases of the task.

For reliability computation in READIE, we used the split-half reliability method rather than calculating Cronbach’s alpha, another commonly used way to estimate internal consistency. Cronbach's alpha calculates the average correlation between all possible pairs of items in a test (Hajjar, 2018). While it provides a comprehensive measure of internal consistency, it has limitations, such as assuming that the order of items is the same across subjects and having a direct dependency on the number of trials (Allen et al., 2004, Parsons et al., 2019, Towers and Allen, 2009). This is a concern when examining the reliability of ERP paradigms, which can have hundreds of trials presented in a different order across participants, and when conducting reliability analyses in an increasing number of trials. Because of this, we employ split-half reliability in the READIE Toolbox, following previous research (e.g., Amir et al., 2023; Morales et al., 2022; Morales et al., 2021; Parsons et al., 2019; Towers and Allen, 2009). Estimating split-half reliability entails randomly dividing the dataset into two halves and then assessing the consistency of results across these splits by estimating the correlation between these halves. Typically, the correlation estimates are refined using the Spearman-Brown prophecy formula (Nunnally Jr., 1970). Cronbach's alpha and split-half reliability are tightly related, as Cronbach’s alpha for all items equals the average of the Spearman-Brown-corrected correlation coefficients derived from every possible split (Towers and Allen, 2009). In addition, while Cronbach’s alpha provides overall reliability estimates for the measurement, split-half analysis can be used to assess the overall reliability when trials are not presented in a fixed order and provide reliability estimates as the number of trials increases. The overall reliability offers a general estimate of the measure's consistency for that sample, while analyzing the increasing number of trials provides insights into the number of trials needed to reach different thresholds of reliability (e.g., acceptable [.60], good [.70–.80], and excellent [.90]; Clayson and Miller, 2017; Meyer et al., 2014). These thresholds provide guidelines for evaluating the reliability estimate of a certain task. Providing reliability estimates in an increasing number of trials informs the determination of minimum trial thresholds for including participants in individual difference analyses and for optimizing trial numbers in future study designs.

This split-half approach has been widely used in the EEG field with adults (e.g., Towers and Allen, 2009) and pediatric populations (e.g., Meyer et al., 2014). Furthermore, studies have employed reliability estimates in increments of trial numbers to determine the optimal number of trials needed for robust reliability assessments of specific ERP components (Morales et al., 2022, Towers and Allen, 2009). Our integration of the split-half approach as a measure of internal consistency into the READIE Toolbox is designed to be intuitive, practical, and user-friendly, with minimal dependencies on external libraries, modules, and software components. It simplifies the analytical process, allows easier adoption by novice and advanced researchers, and facilitates its application in research settings.

### 3.2 Within-subject effect size

Effect sizes quantify the magnitude of differences between groups or conditions and provide an estimate of the data quality (Boudewyn et al., 2018, Delorme, 2023, Kappenman and Luck, 2010). Unlike reliability, which assesses the stability or consistency of individual difference measures, effect sizes for within-subject designs focus on quantifying the magnitude of differences in experimental manipulations. Effect sizes in the context of EEG are often reported between different experimental conditions (e.g., congruent vs. incongruent; errors vs. correct trials, etc.) or compared to a pre-stimulus baseline in within-subjects designs, aiming at detecting specific ERP components (e.g., N2, P3, or ERN). Effect sizes evaluate the impact of the measure of interest on studied processes and can help determine the likelihood of detecting a significant effect if it exists (Luck et al., 2021). Furthermore, effect sizes facilitate power analysis, which aids in determining the appropriate sample size, improving study design, and enhancing study replicability. In addition, given the increasing momentum of utilizing EEG in large developmental datasets, small effects can achieve statistical significance due to the large sample size. Thus, given that sample sizes continue to grow in large developmental studies, effect sizes become increasingly important because they quantify the magnitude of differences.

Effect size indicating experimental manipulations can also be viewed as a sign of data quality. This interrelation is critical because noise can obscure the detection of the manipulation of interest, thereby diminishing the perceived effect size. Here, we use effect size measures to assess data quality and quantify the impact of experimental manipulations. Previous studies have utilized effect size as a metric for data quality comparison. For example, Delorme (2023) used effect sizes to compare different EEG preprocessing parameters and pipelines, indicating the performance of various pipelines. Similarly, Kappenman and Luck (2010) employed this metric to quantify the differences between EEG systems, assessing the impact of different systems on data quality. However, to our knowledge, it has not been used to examine data quality in pediatric populations. In our toolbox, we have built-in within-subject effect size functions to help users quantify the magnitude of differences between conditions within a task as an additional data quality metric.

### 3.3 Standard Measurement Error (SME)

Recently, Standard Measurement Error (SME) has been proposed as a simple and flexible approach to represent the standard error of measurement for ERP measures (Luck et al., 2021). It indicates the spread of values across trials and measures the standard deviation of the sampling distribution for these measurements (Luck et al., 2021). In addition, it provides a measure of variability at the individual level, both at the individual participant level and at the level of individual channels within a participant. Providing a data quality metric at the individual level is a key advantage of SME compared to internal consistency reliability and effect sizes, which offer generalized measures for the entire sample. This individual-level metric facilitates the exclusion of participants or problematic channels. Moreover, SME, closely related to internal consistency reliability, quantifies the degree of change under repeated measurements within the same condition. This enables researchers to quantify differences in data quality between experimental designs, preprocessing, and data analysis parameters. Additionally, it aids in the identification of potential issues that impact data quality, such as detecting environmental noise or identifying bad channels (Luck et al., 2021). Studies have employed SME to evaluate EEG data on both adults and children; for example, Zhang and Luck (2023) observed large differences in SME across different ERP components among college student participants (e.g., P3b, N170, mismatch negativity, N400). In addition, Isbell and Grammer (2022) applied SME calculations to ERP data collected from young children, suggesting that SME can be used to reach decisions about appropriate scoring methods and baseline correction for pediatric EEG data.

Similar to the context-dependent application of internal consistency reliability, it is important to note that SME’s application should be considered depending on the context as well. For example, for tasks that expect varying responses over time or studies involving neurodiverse populations, the use of SME may need to be carefully evaluated, given that their varied responses might indicate learning, developmental changes, or neurodevelopmental differences between groups rather than poor data quality. Therefore, while SME is valuable for assessing data quality in certain contexts, it should be applied with caution and an understanding of its meaning in specific research scenarios. Given these considerations, our tool provides researchers with well-established data quality metrics, including internal consistency reliability and effect sizes, alongside this innovative metric.

### 3.4 Summary of READIE toolbox metrics

In summary, the three metrics discussed—internal consistency reliability, within-subject effect size, and SME—each play a unique role in assessing data quality. Reliability focuses on the internal consistency of a dataset, which is crucial for identifying individual differences. The within-subject effect size quantifies variations between conditions, capturing the brain's response to experimental manipulations and providing information about data quality. SME evaluates the precision of the measurement for each participant, enabling detailed analysis at the individual level. Collectively, these metrics offer a comprehensive framework for understanding different dimensions of data quality, ensuring both the reliability and robustness of the data, informing future research designs, and being a prerequisite to the accurate interpretation of the research findings. Furthermore, considering their distinct implications, it is important to apply these metrics judiciously, aligning them with the most suitable contexts, experimental designs, and research questions.

## 4 Methods

### 4.1 Split-half reliability

To assess the internal consistency of EEG data, we employ the split-half reliability method, a technique widely used in prior EEG research (e.g., Leach et al., 2020; Morales et al., 2022; Towers and Allen, 2009). We utilize participant trial-level data to estimate the overall reliability and identify the minimum number of trials needed to obtain a reliable measure through subsampling. For overall reliability, we first randomly split all trials per condition into two bins, correlate the scores, and then apply the Spearman-Brown formula to estimate reliability. We repeat this process many times (e.g., 1000–5000) to obtain a robust estimate of overall internal consistency reliability. For increasing numbers of trials, we first randomly create a subsample of trials and conduct Spearman-Brown corrected split-half correlations for reliability calculation. We iteratively increase the number of trials (*n*) in the subsamples (e.g., 5–100 in increments of 5) to examine when the reliability estimates reach different thresholds (e.g., acceptable [.60], good [.70–.80], and excellent [.90]; Clayson and Miller, 2017; Meyer et al., 2014). Because estimates depend on which trials are sampled, several iterations are conducted (e.g., 1000–5000) in which the reliability is estimated for each number of trials (*n*). This iterative procedure provides a robust measure as it allows for the split-half randomization to vary across iterations. We utilize the generated distribution of reliability estimates from the iterative procedure to calculate confidence intervals around the reliability estimates for both overall and increasing number of trials. Specifically, the steps of calculating split-half reliability analysis include:

1. Subsampling: A given number of trials (*n*) from each participant and condition is subsampled without replacement.
2. Splitting of Trials: Within each participant, the subsampled *n* trials are randomly divided into two bins.
3. Averaging: The averaged value is calculated in each bin for each participant.
4. Correlating Scores: The average scores are calculated for all participants between the two bins.
5. Applying the Spearman-Brown Formula (Eq. 1): This formula is employed to adjust the observed correlation coefficient between the halves of the test to estimate the correlation coefficient for the full test and calculate the reliability estimate derived from the test (Warrens, 2017).
  (1) $$\begin{array}{c} r_{\mathrm{full}}=\frac{2{(r}_{\mathrm{half}})}{1+r_{\mathrm{half}\;}}\\ \mathrm{Spearman}-\mathrm{Brown}\;\mathrm{Formula} \end{array}$$
  Where:
  $r_{\mathrm{full}}$ is the reliability coefficient of the full-length test.
  $r_{\mathrm{half}}$ is the reliability coefficient of the half-length test.
6. Repeating and Averaging: The entire process is repeated multiple times (e.g., 1000–5000), and the average, as well as the 95 % confidence intervals of the reliability estimates, is calculated. This repetition generates a distribution of reliability estimates, from which the 2.5th and 97.5th percentiles are used to determine the confidence intervals.

### 4.2 Within-subject effect size

To estimate effect sizes, we calculate Cohen's d between conditions (Eq. 2). Cohen's d is a standardized measure that quantifies the difference between the means of two conditions by dividing the mean difference by the pooled standard deviation (Boudewyn et al., 2018).

(2) $$\begin{array}{c} d=\frac{\overset{¯}{X_{1}}-\overset{¯}{X_{2}}}{s}\\ \mathrm{Cohen}\prime s\;d\;\mathrm{Formula} \end{array}$$

Where:

d is the Cohen’s d effect size.

$\overset{®}{X_{1}},\;\overset{®}{X_{2}}$ is the mean of condition 1 and condition 2.

s is the pooled standard deviation.

We follow the same overall and subsampling process for increasing number of trials as described in the internal consistency reliability section. Specifically, the steps of computing within-subject effect size include:

1. Subsampling: A given number of trials (*n*) from each participant and condition is subsampled without replacement.
2. Computing Within-Subject Effect Size: For conditioned data, effect size calculations are conducted compared to the baseline, as well as cross-conditions. Both one-sample t-tests for data within a single condition (compared to the baseline or zero) as well as paired-sample t-tests between different conditions are performed.
3. Repeating and Averaging: The entire process is repeated multiple times (e.g., 1000–5000), and the average, as well as the 95 % confidence intervals of the effect size, is calculated. This repetition generates a distribution of effect size estimates, from which the 2.5th and 97.5th percentiles are used to determine the confidence intervals.

### 4.3 Standard Measurement Error (SME)

For SME estimation, we follow the approach described by Luck et al. (2021). In their work, Luck et al. (2021) distinguished different conditions under which analytic standardized measurement error (aSME) and bootstrapped standardized measurement error (bSME) should be used for data precision measurement. Specifically, aSME is applicable to the time-window mean amplitude because this score remains consistent whether it is obtained from the averaged ERP waveform or by averaging individual single-trial scores (Luck et al., 2021). In contrast, bSME should be used for peak amplitudes or peak latency (Luck et al., 2021). When measuring these scores, the values obtained from averaging individual trials differ from those obtained from the averaged ERP waveform, and the standard errors also differ. Therefore, bSME utilizes bootstrapping with replacement to estimate the standard error of the data. In the READIE Toolbox, we provide calculations for both aSME and bSME for cross-validation purposes. Note that the READIE Toolbox currently only accepts mean amplitude values.

For aSME calculation for each participant, we apply the following equation (Eq. 3). We compute the standard deviation (SD) across trials of a single participant and divide it by the square root of the number of trials (N) for that participant (Eq. 3).

(3) $$\begin{array}{c} SME=\frac{\overset{ˆ}{\mathrm{SD}}}{\sqrt{N}}\\ \mathrm{SME}\;\mathrm{Equation} \end{array}$$

Where:

$\hat{\mathrm{SD}}$ is the estimated standard deviation across trials of a single participant.

N is the number of trials for that participant.

Following the same approach used in Luck et al. (2021) for bSME calculation, we utilize bootstrapping with replacement to estimate the standard error of our data. Specifically, the steps of computing bSME for each participant include:

1. Bootstrapping: New samples are simulated from an existing set of trials through bootstrapping with replacement for each participant.
2. Repeating: The entire bootstrapping process is repeated multiple times (e.g., 1000–5000). Sampling distribution is generated by participant.
3. Computing bSME: The bSME for the participant is the standard deviation (SD) of the sampling distribution.

## 5 Data utilized for illustration

### 5.1 Participants

The dataset utilized in this manuscript to illustrate our toolbox was collected as part of an ongoing longitudinal Khula study of the first 1000 days (see Zieff et al., 2024 for complete longitudinal protocol). Briefly, families participated in three in-lab study visits over their infant’s first two years of life: one between 2 and 5 months of age (Visit 1; mean age: 4.09 months, SD = 0.91, range = 2.07 – 5.82 months), one between 6 and 12 months (Visit 2; mean age: 9.46 months, SD =1.62, range = 6.04 – 12.96 months), and one between 13 and 18 months (Visit 3; mean age: 15.41 months, SD = 1.16, range = 13.04 – 18.68 months). For the purposes of illustrating age-related changes, we excluded infants who are outside the age ranges of 2–5 months (*n* = 3), 6–12 months (*n* = 4), and 13–18 months (*n* = 0) for visits 1, 2, and 3, respectively. The number of participants is reported in the description of each task, as the number of participants differed by task. For all visits, infant electroencephalography (EEG) data were collected. Not all infants provided usable data for all tasks or at all time points.

### 5.2 EEG data acquisition

Description of the EEG data collection can be found in previous reports (e.g., Margolis et al., 2024). EEG data were recorded while infants sat on their caregivers’ laps using high-density (128-channel) HydroCel Geodesic Sensor Nets (Magstim EGI; Whitland, UK). Nets with modified taller (9.3 mm) pedestals designed for improving the inclusion and experience of infants with curly, coiled, and/or coarsely textured hair were used as needed (Mlandu et al., 2024). EEG data were recorded at a sampling rate of 1000 Hz and online referenced to the vertex (channel Cz) via NetStation 5.4 software (Magstim EGI) connected to a Net Amps 400 Series high-input impedance amplifier. Impedances were aimed to be kept below 100 kΩ in accordance with the impedance capabilities of the amplifier. EEG data were collected during a visual-evoked potential (VEP) task, a passive-viewing face processing task, and a Resting State task. All tasks were administered using E-prime 3.0 software (Psychology Software Tools, Pittsburgh, PA) on a Lenovo desktop computer with an external monitor 19.5 in. on the diagonal facing the infant (with monitor approximately 65 cm away from the infant on their caregivers’ lap). During all tasks, an experimenter was seated near the infants to keep them calm and engaged while stimuli were presented.

#### 5.2.1 VEP Task

For the VEP task, a standard phase-reversal VEP was induced using a black and white checkerboard stimulus (1 cm×1 cm squares within the board) alternating every 500 ms for a total of 100 trials. Participant attention was monitored via video and by an assistant throughout data collection and the task was repeated if participants looked away. See Margolis et al., (2024) for a more in-depth description of this task in this sample. For this task, there were 234, 254, and 247 infants for Visits 1–3, respectively.

#### 5.2.2 Face processing task

A passive-viewing face processing task was only collected at visits 2 and 3. Stimuli were images of Model BF – 016 from the Chicago face database (Ma et al., 2015) expressing happy and fearful emotions. At Visit 2, a maximum of 150 trials were presented to each infant, 50 of each condition (happy upright, happy inverted, and fear upright). At Visit 3, a maximum of 100 trials were presented to each infant, 50 of each condition (happy upright and fear upright). Stimuli were presented on a white background. Each face stimulus was presented for 500 ms, with a variable interstimulus interval (ISI) between 500 and 800 ms. Stimuli were presented in blocks of 5 within each condition and condition presentation block was randomized. For this task, there were 218 (Visit 2) and 225 (Visit 3) infants for Fearful facial expressions and 216 (Visit 2) and 223 (Visit 3) infants for Happy facial expressions.

#### 5.2.3 Resting state task

EEG was collected while infants passively viewed a silent 3-minute resting state video consisting of different colorful and engaging clips. The nearby experimenter also engaged the infants with bubbles or another silent toy as needed during the recording sessions. For this task, there were 243, 240, and 258 infants for Visits 1–3, respectively.

### 5.3 EEG data pre-processing

All EEG files were processed using the Harvard Automated Processing Pipeline for EEG, an automated preprocessing pipeline designed for infant EEG data (HAPPE; Gabard-Durnam et al., 2018; Monachino et al., 2022). Version 3.3 of the HAPPE pipeline was run using MATLAB version 2022b and EEGLAB version 2022.0 (Delorme and Makeig, 2004).

For the VEP task, we generated mean amplitudes for the N1 (40–100 ms), P1 (75–130 ms), and N2 (100–230 ms) in the Oz electrode cluster (E70, E71, E75, E76, & E83; see Margolis et al., 2024 for more details). For the face processing task, we generated mean amplitudes for the P1 (60–140 ms), N290 (100–300 ms), and P400 (300–500 ms) for the Fear and Happy upright facial expressions in an extended Oz electrode cluster (E71, E74, E75, E76, E82, E70, E66, E65, E69, E83, E84, E89, & E90) through the generateERPs script (HAPPE; Gabard-Durnam et al., 2018, Monachino et al., 2022). We selected these time windows based on visual inspection and previous research (Bowman et al., 2022, Conte et al., 2020, Peltola et al., 2020, Xie et al., 2019). For the Resting State task, we segmented artifact-free data into contiguous 2-second windows and estimated absolute power to extract delta (2–3.99 Hz), theta (4–5.99 Hz), low alpha (6–8.99 Hz), high alpha (9–12.99 Hz), beta (13–29.99 Hz), and gamma (30–50 Hz) band power across all electrodes using the HAPPE generatePower script (Gabard-Durnam et al., 2018, Monachino et al., 2022). These frequency bands were selected based on previous research (Gabard-Durnam et al., 2019).

## 6 Results

### 6.1 Reliability, effect size, and SME sample results

The READIE Toolbox is an open-source tool written in MATLAB. The READIE Toolbox with a comprehensive user guide and the sample R code for plotting can be accessed here: https://github.com/Bead-Lab/The-READIE-Toolbox-Reliability-Effect-size-And-Data-quality-In-EEG. We developed the tool to facilitate the estimation, per dataset, of reliability, effect size, and standardized measurement error (SME; Luck et al., 2021) with a user-friendly interface. We used data from the VEP task, face processing task, and Resting State task in the previously described longitudinal birth cohort dataset, the Khula Study (Zieff et al., 2024), to demonstrate how to use and interpret outputs from the READIE Toolbox.

### 6.2 Example results from READIE toolbox

The following section showcases the internal consistency reliability, effect size, and SME results with 1000 iterations used for computation of the VEP, face processing and Resting State datasets, outputted from the READIE Toolbox. To consolidate multiple outputs into a single visualization, we utilized a plotting R script to integrate READIE output into the following graphs.

#### 6.2.1 Reliability output

**Fig. 1:**
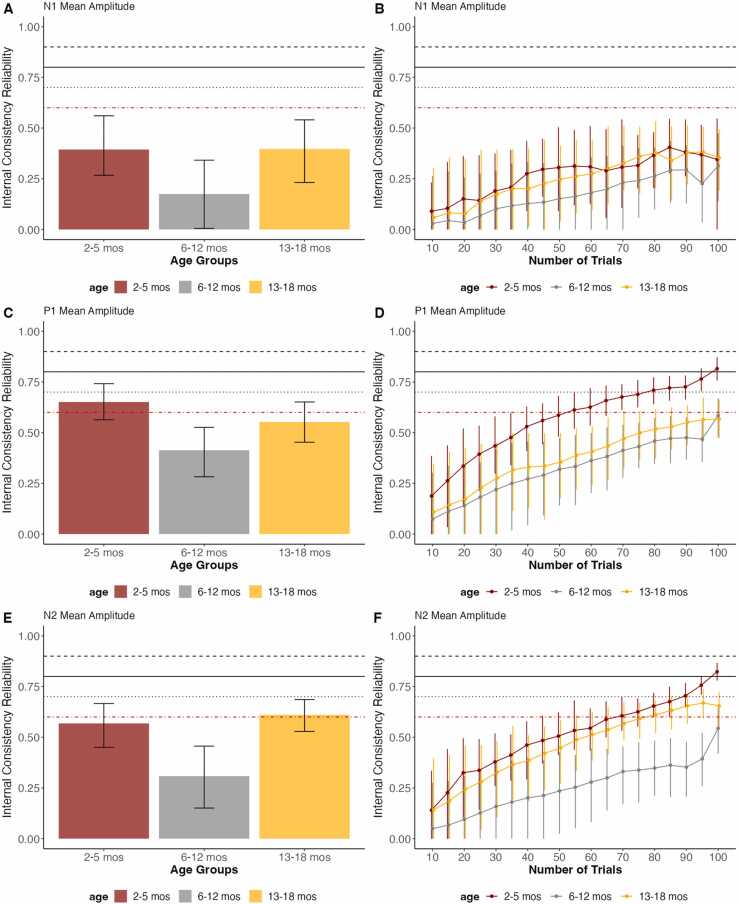
Overall Reliability as well as the reliability in increasing number of trials of VEP data across Age Groups for N1, P1, and N2 Component’s mean amplitudes. The error bars represent 95 % confidence intervals from the resampling distribution. The red dotted line represents the threshold for acceptable data quality (.60), the black solid line represents the threshold for good data quality (.80), and the black dotted line represents the threshold for excellent data quality (.90).

**Fig. 2:**
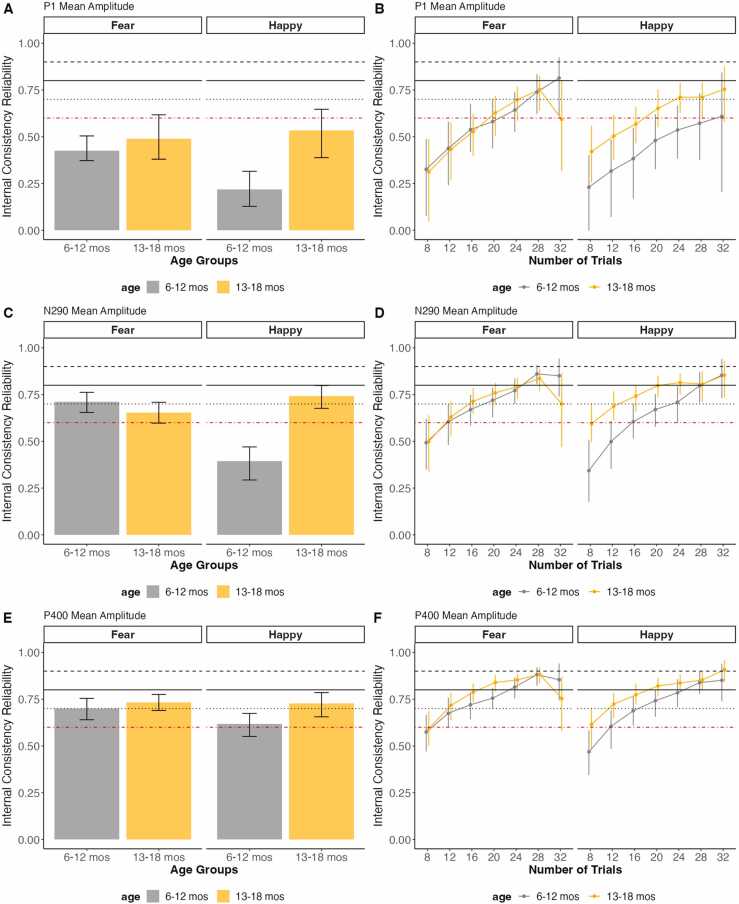
Overall reliability as well as reliability in increasing number of trials of face processing data across three visits for P1, N290 and P400 Components’ mean amplitude. The error bars represent 95 % confidence intervals from the resampling distribution. The red dotted line represents the threshold for acceptable data quality (.60), the black solid line represents the threshold for good data quality (.80), and the black dotted line represents the threshold for excellent data quality (.90).

**Fig. 3:**
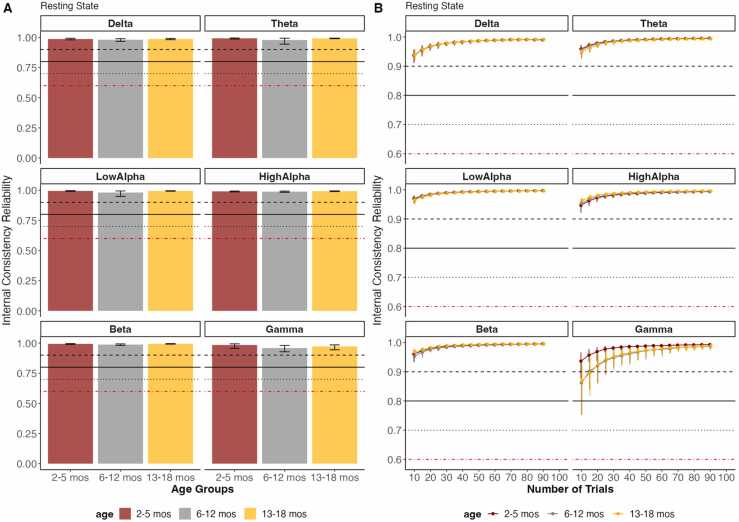
Overall Reliability as well as the reliability in increasing number of trials of Resting State data across three age groups for power in each frequency band. The error bars represent 95 % confidence intervals from the resampling distribution. The red dotted line represents the threshold for acceptable data quality (.60), the black solid line represents the threshold for good data quality (.80), and the black dotted line represents the threshold for excellent data quality (.90).

The READIE Toolbox provides overall and trial-level reliability, as illustrated for the VEP data (Fig. 1), face processing task data (Fig. 2), and Resting State task data (Fig. 3) across the three age groups (2–5 months; between 6–12 months; between 13–18 months). For reliability in increasing trials numbers, the reliability estimates increased as the number of trials increased for all age groups and tasks. Interestingly, the number of trials needed to achieve different thresholds differed by age and the task/measure used. The Resting State task showed the highest internal consistency reliability and achieved excellent reliability with only a few seconds of data. In contrast, the ERP tasks required more trials, with the VEP task requiring the most trials. For the VEP task (Fig. 1), the P1 and N2 components had higher reliability compared to the N1 across age groups. Additionally, for all three ERP components in the VEP task, infants exhibited lower reliability at 6–12 months than at other age groups. For the face processing task (Fig. 3), the N290 and P400 components displayed higher reliability than the P1 component.

#### 6.2.2 Effect size output

**Fig. 4:**
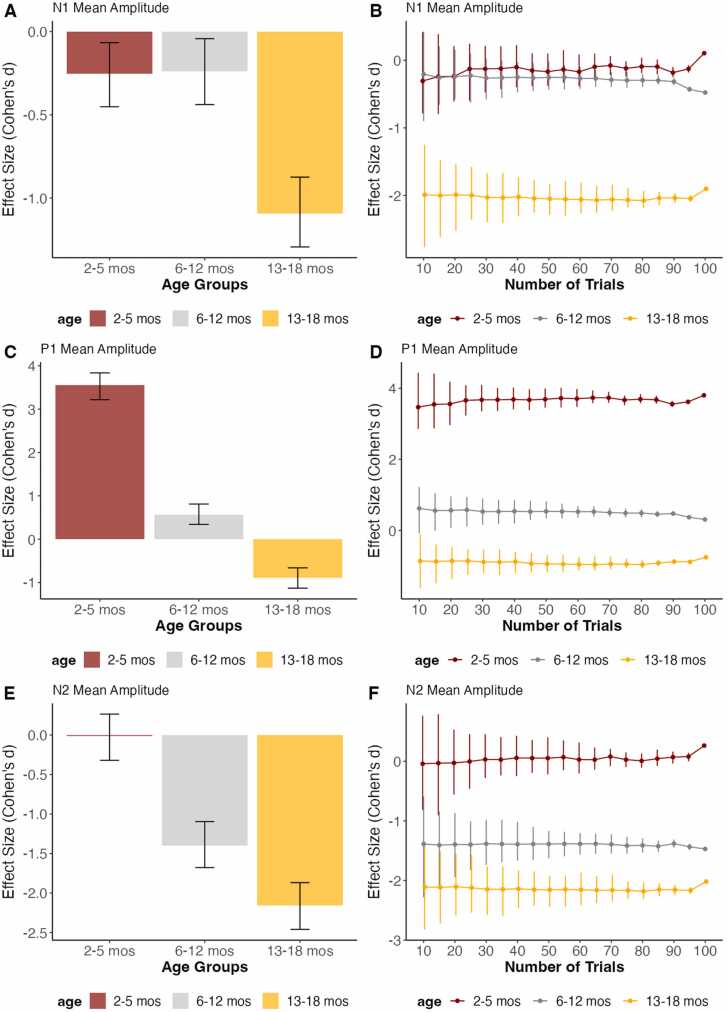
Overall effect size as well as effect size in increasing number of trials of VEP Task data across age groups for N1, P1, and N2 component’s mean amplitude, tested against the pre-stimulus baseline. The error bars represent 95 % confidence intervals from the resampling distribution.

**Fig. 5:**
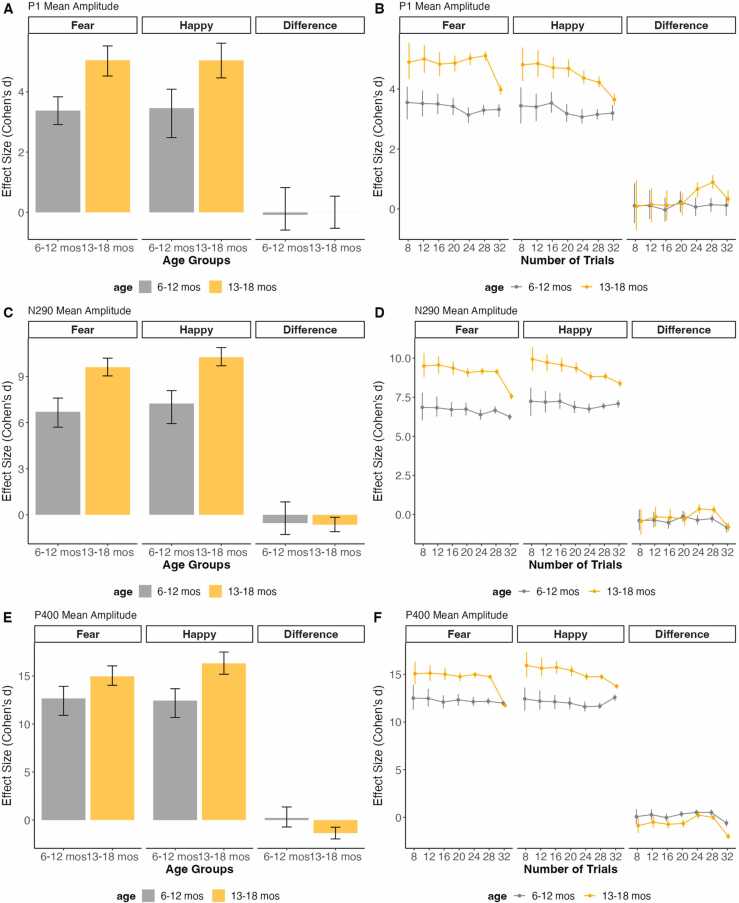
Overall effect size as well as effect size in increasing number of trials of faces processing data across age groups for P1, N290, and P400 component’s mean amplitude, tested against the pre-stimulus baseline. The error bars represent 95 % confidence intervals from the resampling distribution.

The READIE Toolbox provides overall and trial-level effect sizes as a data quality metric. For individual components, the effect sizes represent a comparison between the component to the pre-stimulus baseline. This is illustrated using the VEP data (Fig. 4) and face processing data (Fig. 5) across the different age groups. Across tasks, there was no change in effect size with an increasing number of trials, so all graphs reflected consistent effect sizes across different numbers of trials. Despite the lack of changes with increasing number of trials, the variability around the effect sizes, as reflected by the confidence intervals, was reduced as trials increased. Additionally, given that the order of comparison affects the positive or negative value of effect size, the magnitude of the effect size (how much it differs from 0) is used for comparing in addition to the sign of the effect size. Positive values indicate greater than the pre-stimulus baseline, whereas negative values indicate less than the pre-stimulus baseline. For the VEP task, 13- to 18-month-old infants exhibited a larger effect size compared to the other age groups for the N1 and N2 component. In addition, 2- to 5-month-old infants exhibited a larger P1 component compared to the other age groups. This is in line with the developmental changes in these VEP components (Margolis et al., 2024). Similarly, for the face processing task, older infants had a larger effect size for the P1 and P400 components, but a smaller N290 component relative to the pre-stimulus baseline. In addition, the face processing task allowed us to illustrate the between-condition effect size by contrasting the Happy and Fear conditions based on the facial expression. As shown in Fig. 5, this difference between conditions had a small effect size that was similar across ages and number of trials. We didn’t offer an effect size estimate for the Resting State task, as effect size makes more sense when the task involves differences between groups or conditions.

#### 6.2.3 SME output

**Fig. 6:**
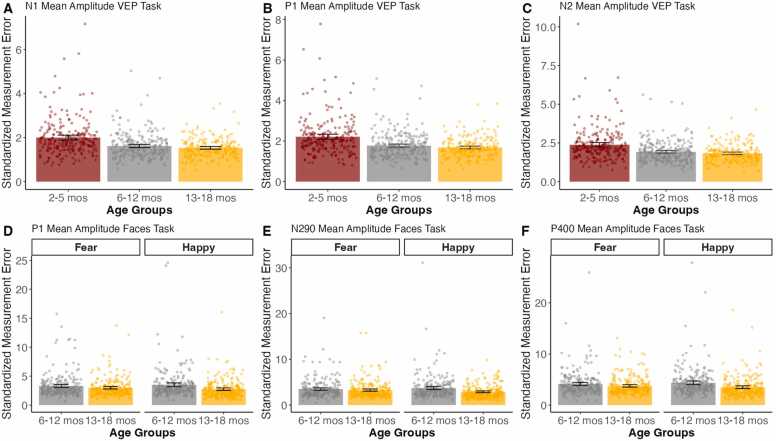
Averaged SME output across age groups for the VEP and face processing data. The error bars represent 95 % confidence intervals of the variability between individuals. Dots represent the individual participants.

The READIE Toolbox also provides SME as an additional data quality metric. Fig. 6 illustrates the summary output of the averaged SME for VEP and face processing tasks. For the VEP task, across all components, the SME was larger for the 2- to 5-month-old infants, compared to 6- to 12-month-old and 13- to 18-month-old infants (*t’s* >= 5.39, *p’s* <.001, d’s =.40–.52). However, there were no differences between 6- to 12-month-old and 13- to 18-month-old infants (*t’s* <= 1.32, *p’s* =.189 −.244, d’s =.09–.10). For the face processing task, there were no condition by age group interactions. However, there were differences by age and condition, such that SME tended to be higher for the 6- to 12-month-old compared to the 13- to 18-month-old infants across conditions for the N290 and P400 (*t* >= 2.15, *p’s* <.033 -, d’s = 0.16–0.26). However, there was not a difference between age groups in the P1 component (*t* = 1.62, *p* =.105, d = 0.12). Across ages, the Happy condition tended to have smaller SMEs than the Fear Condition for all components (*t’s* >= 2.10, *p’s* <.040, d’s = 0.13–0.15). Finally, the individual data points are also shown in Fig. 6 to illustrate that this measure can characterize individual observations, showing wide variability within age groups.

## 7 Developer's notes for the tool

We have integrated our tool with HAPPE (Gabard-Durnam et al., 2018, Monachino et al., 2022), one of the most widely used automated pipelines for pediatric EEG data preprocessing. The HAPPE user interface provides users with the option to have data quality metrics calculated. Additionally, users can utilize plotting functions within our toolbox to easily visualize their data quality metrics output, either in increments of the number of trials or overall, depending on their selection. The updated version of HAPPE (version 5.0) with data quality metrics calculation capabilities can be found here: https://github.com/PINE-Lab/HAPPE.

In addition to the integration of data metrics computation into HAPPE, the scripts can be easily adapted to process data that has been preprocessed with different preprocessing pipelines such as the MADE pipeline (Debnath et al., 2020). Moreover, in addition to utilizing the plotting functions within the READIE Toolbox, we provide sample R code to create publication-ready plots such as the ones used in this manuscript. The READIE Toolbox with a comprehensive user guide and the sample R code for plotting can be accessed here: https://github.com/Bead-Lab/The-READIE-Toolbox-Reliability-Effect-size-And-Data-quality-In-EEG.

## 8 Discussion

Data quality metrics serve as an important foundation for reporting and interpreting meaningful results from scientific research. Given that data quality metrics are an intrinsic quality of the dataset, it is recommended that research papers consistently report them for each study (Clayson and Miller, 2017, Luck et al., 2021). Thus, we developed the READIE Toolbox to facilitate easier computation of data quality metrics, including reliability, effect size, and SME. In addition, we integrated our tool with the HAPPE preprocessing pipeline (Gabard-Durnam et al., 2018, Monachino et al., 2022) to increase its adoption and ultimately facilitate the reporting of these important metrics in the developmental EEG literature.

### 8.1 Reliability, effect size and data quality metrics (SME)

This paper utilized VEP, face processing task, and Resting State task data from a longitudinal sample with 2- to 18-month-olds to showcase the data quality metrics provided by the READIE Toolbox. For VEP dataset reliability, our results suggest that the 6- to 12-month age group exhibited lower reliability across components compared to the 2- to 5-month and 13- to 18-month age groups. This contrasts with other studies with older children, like Morales and colleagues (2022) with 4- to 9-year-old children, which found linear age-related increases in reliability in error monitoring measures. These non-linear changes in reliability could be due to developmental changes and increased instability in those estimates, as well as potential differences in data collection, such as changes in demands for that visit (e.g., addition of the face processing task). In addition, our results showed that the internal consistency reliability differed by the component examined even within a task. For the VEP, the P1 and N2 components displayed higher reliability compared to N1.

For the face processing task, the N290 and P400 components showed higher reliability compared to the P1. This is notable given that when utilizing this task as an individual difference measure, the P1 component for VEP is often the measure that most strongly relates to other outcomes such as prenatal risk factors (Margolis et al., 2024). Similarly, studies examining face processing often utilize the N290 as an individual-difference measure, such as in relation to autism (Gui et al., 2021, Guy et al., 2021, Tye et al., 2022). Additionally, the wider ERP components are more forgiving for mean amplitude calculation than narrower components (e.g., N1, P1). This might relate to our observation that wide components like P400 and N2 have higher reliability, suggesting that window selection (wider or narrower) may impact the reliability of ERP components. For increasing numbers of trials, the face processing task required less trials than the VEP task to achieve acceptable reliability and had higher overall reliability. The VEP task required over 80 trials for most age groups and components, whereas the face processing task only required ∼20 trials for most components and age groups (and even 16 trials for the N290 and P400 components). This difference in reliability might be due to the magnitude of the ERPs, where we observed that ERPs for face processing task were larger compared to those for the VEP task. Additionally, we would like to highlight that the reliability results for the face processing task should be considered as reflecting ERP responses to faces in general, rather than emotion-specific effects (e.g., Fear vs. Happy).

In contrast to the ERPs, measures from the Resting State task all displayed excellent reliability even with minimal data. This replicates previous studies examining the internal consistency reliability of Resting State measures (Hernandez et al., 2024, Leach et al., 2020, Lopez et al., 2023, Troller-Renfree et al., 2021) and highlights their utility as individual difference measures. Importantly, these age-related and ERP component-related differences in reliability indicate that the internal consistency of a dataset is intrinsic and context-dependent (Clayson, 2020, Morales et al., 2022). Thus, data quality metrics should be consistently reported for different contextual factors such as paradigms and populations. For datasets where the data quality does not meet the acceptable threshold, researchers should be cautious when using such data as a measure of individual differences. For individual differences analysis, researchers should consider increasing the length of the task or adjusting the paradigm to reach an acceptable level of internal consistency in the dataset. Future studies can consider assessing the same participants repeatedly within short time windows to ensure enough data is being collected to obtain robust individual-level measures. In addition, data should be collected with tested and reliable EEG systems, under experimental environments that minimize noise, and data collection procedures should be consistent across sessions. Moreover, continuous effort should be made in developing data preprocessing pipelines that reduce artifacts and noise, ensuring data with a high signal-to-noise ratio are used for analysis.

Regarding effect size, we found that the effect sizes followed age-related changes in the ERPs across tasks. This pattern is similar to that observed by Morales and colleagues (2022), where older children showcased higher effect sizes as indicative of age-related changes. However, we found no change in effect size when trial number increased, suggesting that effect sizes can be observed for all age groups with a few trials. This finding is similar to previous studies with ERPs, in which the effect sizes remained of similar magnitude as the number of trials increased (Morales et al., 2022). This suggests that in contrast to reliability estimates, effect size estimates are less impacted by the number of trials. However, note that, as expected, the variability around the average effect size estimates was reduced as the number of trials increased, enhancing the power to detect significant effect sizes. This highlights one of the advantages and importance of reporting effect sizes in the context of large data. Although effects may reach statistical significance given the increased power afforded by a large sample, the effect sizes can help interpret the magnitude of the differences.

For SME, our results showed that, across tasks, the 13- to 18-month age group had smaller SMEs compared to the other age groups, suggesting greater variability in SME among younger infants. Given that SME is a relatively new data quality metric and various factors such as trial number, data collection procedures, and task paradigms can potentially cause differences in SME scores (Luck et al., 2021), we are unable to isolate single factors that cause the differences in SME across age. However, similar to the reliability estimates, it is likely that older children would have higher data quality metrics. Similarly, given the novelty of this data quality metric, we are unable to establish thresholds for acceptable SME. Thus, we hope that our toolbox will facilitate more frequent reporting of SME, which will help us gain a better understanding of threshold SME and better isolate the different factors that cause differences. Interestingly, the age-related differences in SME are not identical to those observed with the reliability analyses, highlighting the unique and complementary role of each of the data quality metrics.

### 8.2 Limitations and future directions

Our study has certain limitations. First, our tool employs a split-half reliability analysis grounded in Classical Test Theory (CTT), which posits that observed variance is a combination of true-score variance and random error variance. This approach is predicated on the assumption that tasks are in parallel form, characterized by equal means, variances, and covariances (Clayson and Miller, 2017, Cronbach, 1975). However, it is crucial to acknowledge that CTT's assumptions may not universally apply, as external factors such as test location and device can introduce systematic errors into the observed scores. To address these limitations, some developers have turned to Generalizability Theory (G Theory), which offers a more nuanced framework for the estimation of measurement error. G Theory distinguishes between systematic and random errors, facilitating the analysis of multiple sources of error for more comprehensive reliability assessments. This approach is advantageous for handling unbalanced designs and isolating multiple facets of measurement error (Clayson and Miller, 2017). The ERA Toolbox is an example of an application that leverages G Theory for these purposes (Clayson and Miller, 2017).

Secondly, in addition to split-half reliability analysis, Cronbach's alpha is a widely used method for evaluating internal consistency, grounded in Classical Test Theory (CTT). Previous research has used Cronbach’s alpha as a way of estimating reliability in EEG studies (e.g. Neuper et al., 2005; Rietdijk et al., 2014). While both split-half reliability and Cronbach's alpha rely on correlations between items to estimate reliability, they do so in different ways. Cronbach's alpha calculates the average correlation between all possible pairs of items in the test, whereas split-half reliability involves dividing the test into two equal halves and calculating the correlation between the scores on these halves under a certain number of iterations. Previous studies have applied Cronbach’s alpha to examine different factors related to internal consistency. For instance, Thigpen et al. (2017) demonstrated that the number of trials can significantly impact ERP internal consistency when measured using Cronbach’s alpha, with interactions noted between the number of trials and factors such as electrode regions and ERP components. This observation is consistent with our results, where we observed variability in internal consistency using the split-half approach across different age groups, components, and paradigms. Additionally, Cronbach’s alpha has its limitations, such as assuming same order of items across subjects and dependency on the number of trials, which has led some to question its suitability for psychophysiological and cognitive-behavioral data (Parsons et al., 2019, Towers and Allen, 2009). Thus, our toolbox employed split-half reliability for internal consistency due to its ability to compute reliability in increasing number of trials and when trials are not presented in a fixed order across participants.

Additionally, our toolbox provides internal consistency reliability, effect size, and SME measures to assess EEG data quality. However, metrics not included in the toolbox, such as the signal-to-noise ratio (SNR), have also been used to evaluate the quality and interpretability of EEG data by distinguishing meaningful signals from noise (e.g., Luck, 2014; Thigpen et al., 2017). Effect size compared with the pre-stimulus baseline and SNR carry similar meanings as both reflect the strength of the signal relative to the noise or baseline. However, there are differences between the two metrics. SNR divides that signal of interest over the noise. In contrast, effect size indicates the strength of the experimental manipulation (signal of interest), calculated based on the standardized difference between two conditions or with the baseline period (zero). Given that data quality and high signal-to-noise are important determinates for data reliability (Clayson, 2020), future work should integrate SNR into the READIE Toolbox to provide insights into the signal clarity of the EEG components and explore the relationship between SNR and effect size.

Also, although the dataset we used is a longitudinal dataset, we treated the data as cross-sectional to illustrate our toolbox and applied the same time windows and frequency bands across age ranges. The goal of our study was to illustrate the data quality metrics provided by the toolbox and show age-related changes in the data quality metrics, rather than provide a detailed description of how these ERP and power measures change across infancy. However, researchers examining the developmental changes of those ERP components or EEG power measures could identify each individual’s peak to more carefully define the time windows (e.g., Margolis et al., 2024) or frequency bands (e.g., Donoghue et al., 2020). In addition, since wider time windows are more forgiving for mean amplitude calculation, components with wider windows might show higher reliability. Further research should investigate how time window selection affects reliability. Furthermore, our measures did not adjust for the previous component (e.g., peak-to-peak analysis), which some researchers perform. Future studies could examine how those adjustments impact the reliability of the measures.

In addition, our toolbox can only be applied to measures for which mean scores or average waveforms are the same as the average of single-trial scores, such as ERPs’ mean amplitude or EEG power. The READIE Toolbox is not recommended for measures for which this is not the case, such as peak amplitude and latency or time-frequency measures involving variability across trials. For instance, the peak latency for an averaged waveform across trials does not equal the mean of peak latency across trials (Luck et al., 2021). This creates computational complications for reliability estimate calculations as it involves averaging across trials and participants. Luck and colleagues (2021) have discussed ways to estimate SME for latency measures through bootstrapping and Morales and colleagues (2022) have estimated the reliability of time-frequency measures. Future versions of the READIE Toolbox could potentially include data quality metrics for these measures, providing researchers with a broader range of tools for evaluating the quality of EEG data.

Furthermore, for the bootstrap calculations of internal consistency reliability and effect size, we adopted an approach consistent with previous research, which typically employs 1000–5000 iterations for bootstrapping (e.g., Morales et al., 2022; Towers and Allen, 2009). However, we have not yet established a specific threshold for the number of iterations that would ensure stable results while optimizing computational efficiency. Moving forward, we aim to explore how the optimal number of iterations could be dynamically tailored for each study, thereby achieving a balance between reliable outcomes and computational speed.

While we highlight the distinct aspects captured by reliability, effect size, and SME, READIE currently lacks a method for comparing across these metrics to understand how variations in one metric may influence another. Although existing literature explores data quality and reliability metrics for EEG data with adults, the results are scarce and often mixed. For example, Clayson et al. (2020) suggest that internal consistency is related to increased effect sizes for the correct-related negativity (CRN), and SME is negatively correlated with subject-level dependability estimated for reward positivity (RewP). Amir et al. (2023) suggest a positive relationship between SME and between-trial standard deviation for RewP. Conversely, Thigpen et al. (2017) found generally non-linear relations between SNR, internal consistency, and effect size for different ERP measurement techniques. These mixed findings indicate that multiple factors can interact with the interrelations among different metrics. Thus, future studies should focus on elucidating these relations to advance our understanding of data quality metrics in EEG research and provide recommendations about combinations of cutoffs or ranges in these metrics.

We underscore the distinct implications of three key metrics: reliability, effect sizes, and SME. Collectively, they provide a framework for evaluating data quality. Yet, it is critical to recognize that there is no one-size-fits-all solution as different studies have unique manipulations and contexts (e.g., clinical populations, age, and conditions). Some metrics are more informative than others depending on the task and context. For example, effect sizes comparing to baseline is influenced by factors like hair length, while comparing between conditions is more suitable for paradigms with robust condition effects. Therefore, we included additional metrics such as internal consistency and the standard error of measurement (SME) in the toolbox, providing estimates of different aspects of data quality.

Finally, for studies that capture changes in state or developmental processes, it is important to balance robust measures that are stable across iterations of testing with measures that are flexible enough to reflect "true" changes in psychological states or developmental profile. It is crucial to seek variability that is explainable by meaningful factors, so this variability is not treated as noise. Therefore, while advocating for the routine reporting of data quality metrics in developmental research, we emphasize the necessity of selectively applying these metrics to best suit the specific needs of the dataset and the experimental design.

## 9 Conclusion

The paper has discussed three data quality metrics: reliability, effect sizes, and SME; highlighting their importance in measuring data quality. Following existing literature that advocates routine reporting of reliability estimates, we aim to facilitate the estimation and reporting of EEG data quality metric analysis by providing the READIE Toolbox that is intuitive, user-friendly to researchers, and integrated with existing preprocessing pipelines. We hope the toolbox will help promote higher standards for reliability and reproducibility in future developmental neuroscience research.

## Funding

This research was supported by grants from the 10.13039/100000865Bill and Melinda Gates Foundation (INV-047884) to LGD and SM and Wellcome LEAP to LGD and KAD.

## CRediT authorship contribution statement

**Michal R. Zieff:** Writing – review & editing, Data curation. Lauren Davel: Writing – review & editing, Data curation. **Cassandra J. Franke:** Writing – review & editing, Data curation. **Cara Bosco:** Writing – review & editing, Data curation. **Ana Sobrino:** Writing – review & editing, Data curation. **Emma T. Margolis:** Writing – review & editing, Data curation. **Sarah A. McCormick:** Writing – review & editing, Data curation. **Alexa D. Monachino:** Software, Methodology, Formal analysis, Writing – review & editing. **Santiago Morales:** Writing – review & editing, Writing – original draft, Visualization, Supervision, Project administration, Methodology, Funding acquisition, Formal analysis, Conceptualization. **Wenyi Xu:** Writing – review & editing, Writing – original draft, Visualization, Methodology, Formal analysis, Conceptualization. **Laurel J. Gabard-Durnam:** Writing – review & editing, Supervision, Project administration, Funding acquisition, Data curation, Conceptualization. **Kirsten A. Donald:** Writing – review & editing, Supervision, Project administration, Funding acquisition.

## Declaration of Competing Interest

The authors declare no conflicts of interest.

## Data Availability

Data will be made available on request.
